# Emotional reactions to the aged future self matter: A 6-month longitudinal study on retirement planning

**DOI:** 10.1093/geroni/igaf122.897

**Published:** 2025-12-31

**Authors:** Dannii Yeung, Edwin Ka Hung Chung

**Affiliations:** City University of Hong Kong, Hong Kong, China (People’s Republic)

## Abstract

The experimental manipulation of exposing the individuals to their aged future selves is found to be effective in increasing retirement planning. However, it is questionable whether the emotional reactions to the aged future-self image play a role in strengthening the future-self continuity. This study therefore aims to address this research gap using a mixed-research method consisting of an experimental manipulation, a 5-day diary study, and a 6-month follow-up assessment. A total of 203 Hong Kong Chinese workers (Mage = 41.01, SD = 11.34) participated in the experiment where they were randomly assigned to future-self (FS) or current-self (CS) conditions. FS participants were presented with their aged-morphed photos, while CS participants viewed their current photos. Following the experiment, a 5-day diary study assessed participants’ plans to improve their future well-being and retirement activities at the 6-month follow-up. Results from the sequential mediation analysis indicated that, compared with CS participants, FS participants experienced more negative emotions towards their future selves at the experiment, resulting in lower perceived closeness to future self during the 5-day diary period and fewer retirement planning activities at the 6-month follow-up [Financial domain: B= -0.02, SE = 0.01 (-0.04, -0.00); Health domain: B= -0.02, SE = 0.01 (-0.05, -0.00); Social domain: B= -0.02, SE = 0.01 (-0.05, -0.00); Psychological domain: B= –0.02, SE = 0.01 (-0.04, -0.00)]. These findings suggest that promoting a positive image of aging can strengthen working adults’ preparation for retirement. A diary approach with positive messages can maintain the connection to the future self and thus facilitate long-term retirement preparation.

